# Auditory Verb Generation Performance Patterns Dissociate Variants of Primary Progressive Aphasia

**DOI:** 10.3389/fpsyg.2022.887591

**Published:** 2022-06-24

**Authors:** Sladjana Lukic, Abigail E. Licata, Elizabeth Weis, Rian Bogley, Buddhika Ratnasiri, Ariane E. Welch, Leighton B. N. Hinkley, Z. Miller, Adolfo M. Garcia, John F. Houde, Srikantan S. Nagarajan, Maria Luisa Gorno-Tempini, Valentina Borghesani

**Affiliations:** ^1^Department of Communication Sciences and Disorders, Ruth S. Ammon College of Education and Health Sciences, Adelphi University, Garden City, NY, United States; ^2^Department of Neurology, Memory and Aging Center, University of California, San Francisco, San Francisco, CA, United States; ^3^Department of Neurology, Dyslexia Center, University of California, San Francisco, San Francisco, CA, United States; ^4^Department of Radiology and Biomedical Imaging, University of California, San Francisco, San Francisco, CA, United States; ^5^Cognitive Neuroscience Center, Universidad de San Andrés, Buenos Aires, Argentina; ^6^Global Brain Health Institute, University of California, San Francisco, San Francisco, CA, United States; ^7^National Scientific and Technical Research Council (CONICET), Buenos Aires, Argentina; ^8^Departamento de Lingüística y Literatura, Facultad de Humanidades, Universidad de Santiago de Chile, Santiago, Chile; ^9^Department of Otolaryngology – Head and Neck Surgery, University of California, San Francisco, San Francisco, CA, United States; ^10^Department of Psychology, Université de Montréal, Montréal, QC, Canada; ^11^Centre de Recherche de l’Institut Universitaire de Gériatrie de Montréal, Montréal, QC, Canada

**Keywords:** primary progressive aphasia, auditory verb generation, semantic processing, lexical processing, errors analysis

## Abstract

Primary progressive aphasia (PPA) is a clinical syndrome in which patients progressively lose speech and language abilities. Three variants are recognized: logopenic (lvPPA), associated with phonology and/or short-term verbal memory deficits accompanied by left temporo-parietal atrophy; semantic (svPPA), associated with semantic deficits and anterior temporal lobe (ATL) atrophy; non-fluent (nfvPPA) associated with grammar and/or speech-motor deficits and inferior frontal gyrus (IFG) atrophy. Here, we set out to investigate whether the three variants of PPA can be dissociated based on error patterns in a single language task. We recruited 21 lvPPA, 28 svPPA, and 24 nfvPPA patients, together with 31 healthy controls, and analyzed their performance on an auditory noun-to-verb generation task, which requires auditory analysis of the input, access to and selection of relevant lexical and semantic knowledge, as well as preparation and execution of speech. Task accuracy differed across the three variants and controls, with lvPPA and nfvPPA having the lowest and highest accuracy, respectively. Critically, machine learning analysis of the different error types yielded above-chance classification of patients into their corresponding group. An analysis of the error types revealed clear variant-specific effects: lvPPA patients produced the highest percentage of “not-a-verb” responses and the highest number of semantically related nouns (production of *baseball* instead of *throw* to noun *ball*); in contrast, svPPA patients produced the highest percentage of “unrelated verb” responses and the highest number of light verbs (production of *take* instead of *throw* to noun *ball*). Taken together, our findings indicate that error patterns in an auditory verb generation task are associated with the breakdown of different neurocognitive mechanisms across PPA variants. Specifically, they corroborate the link between temporo-parietal regions with lexical processing, as well as ATL with semantic processes. These findings illustrate how the analysis of pattern of responses can help PPA phenotyping and heighten diagnostic sensitivity, while providing insights on the neural correlates of different components of language.

## Introduction

Primary Progressive Aphasia (PPA) is a clinical syndrome characterized by progressive deterioration of speech and/or language abilities due to gradual neurodegeneration of specific left-hemisphere networks (Mesulam, 1987; Gorno-Tempini et al., 2004). The neurobiological basis of this rare clinical picture is the toxic deposition of proteins (Spinelli et al., 2017). Patients, relatively young compared to senile dementia of Alzheimer type, often come to medical attention complaining about generic word finding problems (e.g., “*sometimes I can’t remember words*”). Accurate clinical diagnosis and premortem neuropathological prediction can be achieved only thanks to the coordinated work of a multidisciplinary team of neurologists, neuropsychologists, and speech-language therapists, combining clinical, neuroanatomical, genetic, and biomarker evidence (Tee and Gorno-Tempini, 2019).

Three variants are recognized, associated with specific cognitive, behavioral, and anatomopathological correlates (Gorno-Tempini et al., 2011). Neurodegeneration originates in distinct epicenters and spreads along specific brain circuits determining the clinical profile observed (Seeley et al., 2009; Brown et al., 2019). First, patients with the logopenic variant of PPA (or lvPPA) present with prominent anomia (i.e., word-finding difficulties) in the context of spared motor speech and semantic processing (Gorno-Tempini et al., 2008). The deficits manifested by these patients can be traced back to phonological disorder and/or reduced auditory verbal short-term memory (Henry et al., 2016; Leyton et al., 2017). Atrophy appears to originate in left temporo-parietal areas, specifically posterior temporo-parietal junction (TPJ; Migliaccio et al., 2009; Win et al., 2017; Lukic et al., 2019). In the overwhelming majority of cases, the pathology is Alzheimer’s disease (AD; Bergeron et al., 2018). Overall, a growing body of research is highlighting lvPPA heterogeneity above and beyond disease severity, linking differences in clinical profiles to atrophy origin and distribution (Louwersheimer et al., 2016; Ramanan et al., 2020).

Second, semantic deficits, such as poor performance on confrontation naming and single word comprehension, are the hallmark of the semantic variant of PPA (svPPA or semantic dementia, SD; Warrington, 1975; Snowden et al., 1989). The core deficit appears to be a generalized loss of conceptual knowledge (Bozeat et al., 2000; Luzzi et al., 2007; Goll et al., 2010; Piwnica Worms et al., 2010) and atrophy affects the anterior temporal lobe (ATL) bilaterally (yet often asymmetrically) (Hodges et al., 1992; Brambati et al., 2009; Kumfor et al., 2016). In up to 88% of cases the underlying pathology is TDP-type C (Rohrer et al., 2010; Josephs et al., 2011; Snowden et al., 2011; Whitwell and Josephs, 2012; Borghesani et al., 2020a). Finally, the non-fluent/agrammatic variant of PPA (or nfvPPA) is associated with agrammatism and/or motor speech impairments (i.e., apraxia of speech or dysarthria) (Ogar et al., 2007; Mesulam et al., 2009; Utianski et al., 2018). In these patients, atrophy appears to start in cortical and subcortical left posterior frontoinsular regions (Nestor et al., 2003; Grossman et al., 2013; Mandelli et al., 2016, 2018) and the majority of cases are associated with FTLD-TAU (Josephs et al., 2006; Santos-Santos et al., 2016; Spinelli et al., 2017).

Given the growing body of research indicating that each clinical variant is associated with different probabilities of neuropathological changes and specific language network vulnerabilities, an (early) diagnosis has important implications for patient management (e.g., which treatments and clinical trials should be recommended). Moreover, a detailed phenotyping of the different syndromes provides a unique opportunity to study the neurobiological basis of different language processes. Decades of clinical research indicate that PPA patients differ not only in the kind of task that would elicit difficulties, but also in the kind of error produced. For instance, in connected speech samples one can expect disfluencies due to word finding pauses, and phonological errors in lvPPA (Petroi et al., 2014, 2020), lexical retrieval deficits but spared speech production in svPPA, and phonetic and syntactic errors in nfvPPA (Wilson et al., 2010b; Ash et al., 2013). These findings suggest that, when asked to perform the same language task, patients with different PPA variants might all fail yet for syndrome-specific reasons. Specifically, lvPPA patients’ performance might be mainly driven by access to phonological word form or lexical retrieval demands (e.g., Henry et al., 2016), while svPPA patients might be primarily influenced by lexical factors [e.g., familiarity of words (Rogers et al., 2015)], and performance in nfvPPA patients might reflect speech planning/execution and/or syntactic demands (Wilson et al., 2010a). Thus, while looking at percentage correct in a given language task might not be sufficient to delineate the three variants, examining error patterns could allow proper syndromic classification (Dalton et al., 2018). Moreover, PPA variants provide invaluable insights on the neurocognitive correlates of different components of the language system directly relating phonological processing to temporo-parietal structures, semantic processing to ventro-temporal regions, selection, and execution of speech as well as morphosyntactic processing to frontal networks.

Here, we set out to investigate whether performance on a single language task, requiring both processing of auditory input (a noun) and generating verbal output (a verb) at multiple linguistic levels, would be able to dissociate PPA variants based on their error patterns. We analyzed outcomes from an auditory noun-to-verb generation task, which taxes phonological processes (for sublexical analysis of the input), lexical-semantic processes (for access to relevant word category and conceptual knowledge), and motor speech skills (for planning and execution of oral responses). In particular, the verb-generation task requires retrieval of and selection from an array of possible noun-verb combinations, with responses varying along multiple lexical-semantic axis (e.g., semantically impoverished “light” verbs, e.g., go vs. semantically rich “heavy” verbs, e.g., run; see Gordon and Dell, 2003). We hypothesized that all PPA variants would be impaired with respect to healthy controls (HC), with variant-specific error patterns allowing dissociation of the three variants. We also predicted that task performance will be associated with critical features of the stimuli and key neuropsychological characteristics of the patients.

## Materials and Methods

### Participants

A total of 104 volunteers were recruited through the University of California, San Francisco (UCSF) Memory and Aging Center: 21 lvPPA patients (13 female, 63.59 ± 7.61 years old), 28 svPPA patients (17 female, 68.95 ± 6.19 years old), 24 nfvPPA patients (16 female, 67.87 ± 7.23 years old), and 31 age-matched healthy controls (19 female, 73.04 ± 6.1 years old). All patients were English native speakers and met current, published criteria for PPA as determined by a team of clinicians based on a detailed medical history, comprehensive neurological and standardized neuropsychological and language evaluations (Gorno-Tempini et al., 2011). Healthy controls (HCs) were recruited from the UCSF healthy aging cohort, a collection of participants with normal cognitive and neurological exam and MRI scans without clinically evident strokes. All HCs had no psychiatric symptoms or cognitive deficits [i.e., Clinical Dementia Rating (CDR) = 0, and Mini Mental-State Examination (MMSE) ≥28/30]. Demographic and neuropsychological data, showing the expected group differences, are shown in Table 1. Each participant signed informed consent documents in accordance with the Declaration of Helsinki and the study was approved by the UCSF Committee for Human Research.

**TABLE 1:** Participants demographic and neuropsychological characteristics.

	**Controls**	**lvPPA**	**svPPA**	**nfvPPA**
**Demographic**				
***N***	31	21	28	24
Age, mean (SD)	73.0 (6.1)	63.6 (7.6)*A	69.0 (6.2)	67.9 (7.2)*
Education, mean (SD)	17.4 (1.9) (*n* = 30)	16.7 (2.7) (*n* = 20)	17.9 (2.9) (*n* = 28)	16.2 (2.7) (*n* = 24)
Sex, n female	19 (61.3%)	13 (61.9%)	17 (60.7%)	16 (66.7%)
Handedness, n right	21 (72.4%) (*n* = 29)	18 (85.7%) (*n* = 21)	25 (89.3%) (*n* = 28)	19 (79.2%) (*n* = 24)
MMSE (max 30)	29.3 ± 0.7 (*n* = 21)	22.2 ± 4.4 (*n* = 20)*C	23.9 ± 3.9 (*n* = 28)*C	27.5 ± 2.4 (*n* = 24)*
CDR score	0.0 ± 0.0 (*n* = 22)	0.6 ± 0.2 (*n* = 21)*	0.8 ± 0.3 (*n* = 28)*	0.3 ± 0.2 (*n* = 24)*A,B
CDR Box score	0.02 ± 0.1 (*n* = 22)	3.5 ± 1.6 (*n* = 21)*	4.2 ± 2.0 (*n* = 28)*	1.0 ± 1.0 (*n* = 24)*A,B
**Language production**				
Boston (object) naming test (15)	14.9 ± 0.3 (*n* = 21)	10.4 ± 3.9 (*n* = 20)*C	5.5 ± 3.9 (*n* = 28)*B,C	13.7 ± 2.7 (*n* = 24)
Phonemic (D-letter) fluency	17.4 ± 5.3 (*n* = 21)	8.4 ± 4.0 (*n* = 20)*	8.7 ± 4.2 (*n* = 28)*	7.0 ± 4.3 (*n* = 24)*
Semantic (animal) fluency	23.0 ± 4.4 (*n* = 26)	9.8 ± 6.4 (*n* = 20)*	9.3 ± 5.7 (*n* = 28)*C	13.7 ± 6.0 (*n* = 24)*
WAB repetition total (100)	99.0 ± 1.0 (ND)	73.6 ± 10.2 (*n* = 20)A,C	91.5 ± 4.8 (*n* = 27)	91.4 ± 9.0 (*n* = 24)
Language comprehension				
PPVT (16)	15.6 ± 0.5 (ND)	14.2 ± 2.2 (*n* = 20)	8.8 ± 4.2 (*n* = 28)B,C	15.1 ± 1.2 (*n* = 23)
WAB comprehension total	99.0 ± 2.0 (ND)	77.4 ± 14.2 (*n* = 19)C	83.0 ± 8.8 (*n* = 27)C	87.4 ± 4.0 (*n* = 24)
**Auditory sentence-picture matching (%)**	98.6 ± 1.8 (ND)	90.2 ± 9.7 (*n* = 19)	96.2 ± 4.6 (*n* = 22)	92.0 ± 14.1 (*n* = 22)
**Working memory/executive functions**				
Digit span backwards	5.7 ± 1.2 (*n* = 21)	3.0 ± 1.2 (*n* = 20)*A	4.6 ± 1.4 (*n* = 28)*	3.9 ± 1.2 (*n* = 24)*
Modified trails (total time)	23.1 ± 11.3 (*n* = 19)	86.2 ± 37.0 (*n* = 19)*	46.0 ± 28.2 (*n* = 27)*B	48.3 ± 27.2 (*n* = 24)*B
Modified trails (# of correct lines)	12.6 ± 4.3 (*n* = 19)	9.6 ± 5.0 (*n* = 19)*A,C	13.4 ± 2.7 (*n* = 27)	13.4 ± 2.9 (*n* = 24)
Design fluency (# of correct designs)	13.0 ± 3.2 (*n* = 21)	5.9 ± 2.5 (*n* = 19)*	7.5 ± 3.5 (*n* = 28)*	7.2 ± 2.2 (*n* = 24)*
Visuospatial function and memory				
Benson figure copy (17)	15.3 ± 0.8 (*n* = 21)	14.0 ± 3.1 (*n* = 20)	15.5 ± 0.9 (*n* = 26)	15.3 ± 0.7 (*n* = 22)
VOSP number location (30)	9.1 ± 1.0 (*n* = 21)	7.8 ± 2.3 (*n* = 19)	9.0 ± 1.4 (*n* = 28)	8.9 ± 1.5 (*n* = 24)
Benson figure recall (17)	12.6 ± 2.6 (*n* = 21)	6.8 ± 4.6 (*n* = 20)*C	6.5 ± 4.9 (*n* = 26)*C	11.8 ± 2.6 (*n* = 20)
**Verbal memory**				
Digit span forwards	7.1 ± 1.3 (*n* = 21)	4.6 ± 0.9 (*n* = 20)*A	6.3 ± 1.1 (*n* = 28)	5.2 ± 1.2 (*n* = 24)*A
CVLT-SF trials 1- 4 (40)	29.8 ± 3.4 (ND)	14.1 ± 6.8 (*n* = 19)C	16.4 ± 6.6 (*n* = 28)C	24.1 ± 6.0 (*n* = 24)
CVLT-SF 30 sec free recall (10)	8.0 ± 1.1 (ND)	3.4 ± 3.0 (*n* = 21)C	2.9 ± 2.9 (*n* = 28)C	6.8 ± 1.9 (*n* = 24)
CVLT-SF 10 min free recall (10)	7.5 ± 1.3 (ND)	3.1 ± 3.0 (*n* = 19)C	1.4 ± 2.3 (*n* = 28)B,C	6.3 ± 2.3 (*n* = 24)
**ABRS reading and spelling**				
Reading regular words (18)	18.0 ± 0.0 (ND)	16.9 ± 1.5 (*n* = 18)	16.8 ± 2.5 (*n* = 24)	17.1 ± 1.6 (*n* = 22)
Reading irregular words (18)	17.8 ± 0.4 (ND)	14.7 ± 2.3 (*n* = 18)	13.3 ± 3.5 (*n* = 24)	16.3 ± 2.8 (*n* = 22)
Reading pseudowords (18)	16.6 ± 1.0 (ND)	12.3 ± 3.7 (*n* = 17)	14.6 ± 3.7 (*n* = 24)	12.3 ± 4.8 (*n* = 21)
Spelling regular words (18)	9.6 ± 0.6 (ND)	8.1 ± 1.5 (*n* = 17)	8.6 ± 1.3 (*n* = 24)	8.4 ± 1.8 (*n* = 22)
Spelling irregular words (18)	9.1 ± 1.0 (ND)	4.1 ± 3.3 (*n* = 17)	4.1 ± 2.5 (*n* = 24)	6.6 ± 2.4 (*n* = 22)
Spelling pseudowords (18)	9.2 ± 0.7 (ND)	6.7 ± 3.3 (*n* = 17)	7.9 ± 1.9 (*n* = 24)	7.9 ± 1.9 (*n* = 22)

Values shown are mean (standard deviation). Significant differences in performance between groups are indicated by superscripts A, B, and C, for svPPA, lvPPA, and nfvPPA, respectively (p < 0.05; Kruskal and Duncan’s tests). Asterisks indicate significant differences for the PPA groups relative to HC (* for HC where p < 0.05 and ** where p < 0.001). MMSE, Mini Mental State Exam (Folstein et al., 1975); CDR, Clinical Dementia Rating (Morris, 1997); PPVT, Peabody Picture Vocabulary Test (Dunn, 1959); WAB, Western Aphasia Battery (Kertesz, 1982); VOSP, Visual Object and Space Perception Battery-UCSF version; CVLT-SF, California Verbal Learning Test-UCSF version. Normative data (ND) were used for the following scores: The WAB Repetition and Comprehension Total (scores extracted from the WAB-R manual), Syntax Comprehension [Wilson et al., 2010a,b; scores extracted from a normative sample in our center, published in Lukic et al. (2019)], ABRS Reading and Spelling scores [scores extracted from a normative sample in our center, published in Europa et al. (2020)], PPVT (scores extracted from a normative sample in our center with mean age: 67.7 ± 4.1 and education: 18.0 ± 1.2), and CVLT (scores extracted from a normative sample in our center with mean age: 54.7 ± 14.1 and education: 16.7 ± 2.2). Due to a paucity of data in a WAB subtest (Yes/No Questions), WAB Comprehension Total scores for patients were computed using percentage correct scores across only two subtests (Auditory Word Recognition, Sequential Commands) instead of three.

### Behavioral Data Acquisition

**FIGURE 1:**
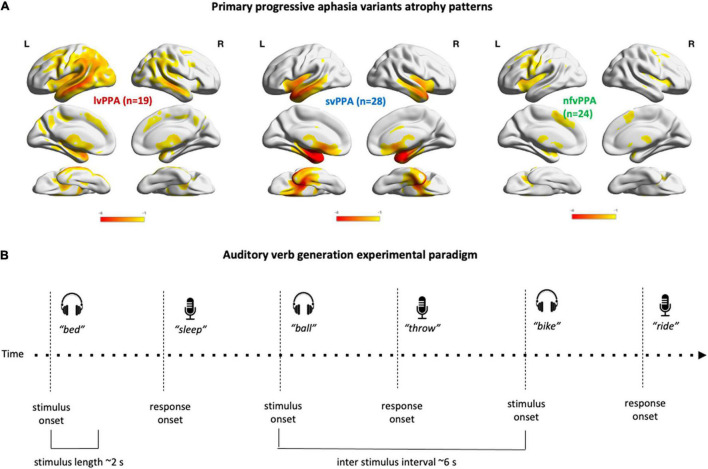
PPA variants atrophy patterns and experimental paradigm. (A) Render illustrating the results of the voxel-based morphometry analysis identifying regions of gray matter volume loss in the three PPA variants relative to healthy controls. (B) Schematic representation of the experimental auditory verb generation task. After each stimulus presentation, a 4 s interval preceded the next stimulus onset.

All participants underwent neuropsychological testing with a comprehensive battery of language, memory, visuospatial, executive functions, and behavior that has been described extensively (Kramer et al., 2003; Europa et al., 2020). Participants performed an experimental auditory verb generation task (Figure 1B). At the beginning of every trial, they were prompted with a noun and instructed to respond with an associated verb. Auditory stimuli included 50 nouns presented twice each in randomized order (Table 2) with an inter-stimulus-interval of 6 s (Petersen et al., 1988). Stimuli varied in concreteness (e.g., “ball” versus “music”), familiarity (e.g., “store” versus “knob”), and imageability (e.g., “car” versus “story”). E-Prime^1^ was used to deliver the stimuli via headphones and verbal responses were spoken into a microphone and recorded for post-processing analysis (Findlay et al., 2013; Hinkley et al., 2020). The task was performed during MEG recordings as part of a large protocol studying language processing dynamics in PPA patients (Borghesani et al., 2020b,^2021^), however, the current study focused on fine-grained analysis of the behavioral performance during the verb generation task.

**TABLE 2:** Psycholinguistic characteristics of the stimuli.

	Nouns	Range
*N*	50	
# of letters	4.76 (1.05)	3–8
# of syllables	1.32 (0.47)	1–2
# of phonemes	3.62 (1.0)	2–6
Frequency COCA (log)	6.02 (1.2)	3.4–8.3
Semantic neighborhood	171.4 (84.3)	28.7–362.9
Concreteness	6.26 (0.6)	3.78–6.88
Familiarity	6.23 (0.5)	4.49–6.94
Imaginability	6.35 (0.6)	3.81–6.92
Age of acquisition	2.51 (0.6)	1.49–4.17
ASI – HC	61.67 (19.31)	28.3–98.4
CSI – HC	9.40 (13.69)	1.1–61
Entropy – HC	1.55 (0.65)	0.1–2.7

*Stimuli consisted of 50 nouns. Values shown are mean (standard deviation), minimum and maximum. Glasgow Concreteness, Familiarity, Imaginability, and Age of Acquisition were extracted from the South Carolina Psycholinguistic Metabase [SCOPE; based on Scott et al. (2019) norms]. Frequency was extracted from the Corpus of Contemporary American English (COCA; Davies, 2008). Semantic neighborhood (N50) was extracted based on taxonomic similarity (Reilly and Desai, 2017).*

Three raters, blind to diagnostic group, independently coded each response as a related verb (e.g., “*ball*” = “*throw*”), not-a-verb (e.g., “*ball*” = “*baseball*”), unrelated verb (e.g., “*ball*” = “*eat*”), or missing. Discrepant judgments were discussed with other authors. Only related verb responses were considered correct. Generally speaking, phrasal verbs (e.g., “*take out/away*” for trash) were accepted as accurate responses. However, based on the healthy control responses and the opinions of the three Native English speakers acting as raters, we decided to reject “*to be careful*” for knife or scissor, and to “*to go in*” for house, while “*to do*” for laundry was accepted. For patients whose responses contained articulatory errors (e.g., “lif-life-ted” to a noun “candle”) or phonological paraphasias (e.g., “seep” instead “sleep”), interpretability and accuracy of response had to be agreed upon by the raters after a thorough review. The articulatory errors were considered missing errors, whereas interpretable phonological paraphasias were considered correct (following Thompson et al., 2012).

The data collected in the healthy control cohort were used to compute an index of overall verb generation agreement for each noun (i.e., H-index or response entropy), following (Snodgrass and Vanderwart, 1980; Kurland et al., 2014): $E=\sum_{i=1}^{k}P\,i*l\,o\,g\,2\,\left(1/P\,i\right)$, where *k* is the number of different verbs generated for each noun and *Pi* is the proportion of subjects generating each verb. This index quantifies the amount of uncertainty associated with each noun by considering both the number of different verbs generated and the proportion of participants generating them. Thus, entropy increases as agreement decreases: for instance, an entropy value of 0.0 would indicate perfect agreement (i.e., all participants generated the exact same verb for a given noun), while a value of 1.00 indicates that participants generated, for a given noun, two verbs with equal frequency [a similar, related, alternative is to assess the spread (i.e., tailedness)] of the distribution of possible responses with measures such as the kurtosis of the frequencies as done in Barch et al. (2000). Accounting for both number and relative distribution of alternative answers, entropy conflates both retrieval demands (*how strongly related is this noun to a verb?*) and competition demands (*how many verbs are commonly associated with this noun?*) in one measure. To assess the potentially differential impact of the two variables on patients’ performance, we also computed two additional measures. Retrieval demands, i.e., the strength of the noun-verb association, were operationalized as the percentage of subjects providing the most common response (hereafter, *Association Strength Index* or *ASI*): the more a noun and verb are semantically associated, the higher the ASI. Competition demands, i.e., the selection constraints associated with a given noun, were operationalized as the ratio between the percentage of subjects providing the most common response and the second most common one (hereafter, *Competition Strength Index* or *CSI*): the wider the gap between the first and second most common verb, the higher the CSI. It should be noted that there are doubts as to whether agreement (ASI) and ratio (CSI) are really good operationalizations of, respectively, association strength and competition demands (see Snyder and Munakata, 2008 for a discussion). However, we sought to adopt measures that had previously been used in similar studies to allow a direct comparison of our findings.

Prior to any further qualitative analyses, behavioral performance (percentage of correct response) was used to determine, separately for the patients and HC cohorts, possible outliers, i.e., participants falling outside of 2 standard deviations from the respective group average. Thus, two HCs (percentage of correct 63 and 68) and two lvPPA (percentage of correct 7 and 8) were removed from further analyses leaving a total sample of 100 participants (HC = 29, svPPA = 28, lvPPA = 19, nfvPPA = 24).

### Behavioral Data Analysis

First, we compared the four cohorts in terms of overall accuracy with ANOVA, and assessed group differences with *post hoc* analysis with Tukey’s Test. Then, a second ANOVA was used to compare the three variants in terms of their error patterns (3 groups × 3 error type), and *post hoc* Tukey’s Test was performed to evaluate pairwise differences. Reaction times are considered a rather uninformative measure in this context, as they will depend not only on variables influencing noun processing (and associated verb retrieval) but also on characteristics of the sound that were not controlled for. However, we compared the three variants with a 3-way ANOVA to assess whether any statistical difference was noticeable (with *post hoc* Tukey’s Test to evaluate pairwise differences). Given that stimuli were presented twice, we also computed and compared (with two new ANOVAs) the percentage of consistent errors (i.e., out of the 50 pairs of stimuli, how often both were responded incorrectly) and the percentage of consistent error type (i.e., out of all the error pairs committed, how often both were of the same type).

Second, to classify patients into one of the three variants a non-parametric supervised machine learning algorithm was used – a decision tree classifier (with a maximum distance between the root and any leaf of 2) implemented in Scikit-Learn (Pedregosa et al., 2011)^2^. Significance level was obtained using a permutation test, empirically estimating the random distribution of chance level.

Third, we investigated the cognitive correlates of patients’ error patterns by conducting exploratory by-items analysis (aiming at detecting the effect of stimuli psycholinguistic characteristics) as well as by-subjects analysis (aiming at detecting the effect of patients’ neuropsychological profiles). To assess whether performance relates to specific properties of the nouns, percentage correct scores were pooled across subjects (variant by variant) and correlated with three psycholinguistic variables known to affect, respectively, semantics, lexical, and word form aspects of word processing (Graves et al., 2007; Wilson et al., 2009): semantic neighborhood density [based on taxonomic similarities by Reilly and Desai (2017)], word frequency (the log COCA frequency), and word length (the number of phonemes). Similarly, correlations between retrieval demands (ASI), selection demands (CSI), and overall agreement (entropy) and task performance were conducted in order to examine effects of cumulative noun-verb association demands on response accuracy. Finally, we selected three neuropsychological measures that are commonly administered across patients to assess cognitive functioning (word comprehension, lexical retrieval, and executive function), which are thought to be distinctively affected in the three PPA cohorts: PPVT (e.g., Borghesani et al., 2020b), ratio of phonemic and semantic fluency (e.g., Staffaroni et al., 2021), and modified trails total time (e.g., Brambati et al., 2015). Correlations were Bonferroni-corrected for multiple comparisons (6 by-item and 3 by-subject).

Fourth, we investigated the related verb responses produced by the three variants to evaluate the weight of lexical-semantic aspects. First, we quantified the dispersion (i.e., distribution) of responses. For each noun and for each cohort, we computed the number of unique verbs produced. Second, for each subject, we examined the percentage of related verb responses containing so-called “light” verbs (e.g., *take*), those core predicates (or primitives) often used as auxiliaries (e.g., *have*), frequently appearing in idioms (e.g., *take over*), seen in the linguistic literature as sharing features of closed class words (i.e., function words). Following previous literature (Breedin et al., 1998; Kim and Thompson, 2004), we predetermined a list of light verbs (*be, bring, come, do, get, give, go, have, make, move, put, see, take*, and *use*) and counted their frequency of occurrence.

Lastly, we characterized the not-a-verb responses by (1) counting the number of simple repetitions of the noun form (e.g., “*ball*” in response to “*ball*”) and (2) computing the semantic similarity between a produced noun and the input noun (e.g., “*baseball*” in response to “ball”). Semantic similarity was computed as the cosine similarity between the two nouns embedding as constructed with a natural language processing algorithm pre-trained on Google News (word2vec, as available via https://pypi.org/project/gensim/). Two nouns were counted as semantically similar if their cosine similarity was higher than the arbitrary threshold of 0.3 (as an example, *“dog-wolf*” similarity is 0.4, “*dog-cloud*” similarity is 0.06), which lies at the median value for all responses across all cohorts (all patients 0.34, lvPPA = 0.29, svPPA = 0.34, nfvPPA = 0.39).

All the analyses described in this section were conducted with in–house python scripts relying on numpy^3^, scipy^4^, and statsmodels^5^.

### Imaging Data Acquisition and Analysis

To delineate the atrophy map for each variant, T1-weighted images were acquired for 70 patients with sequences, previously described, on either 3T (*n* = 42, Mandelli et al., 2014), or 4T (*n* = 28) systems equipped with a standard quadrature head coil. MRI scans were acquired within 1 year of each visit and in each case the first available image was used for analysis. Images were processed using Statistical Parametric Mapping (SPM12, Wellcome Trust Centre for Neuroimaging, London, United Kingdom) running under Matlab R2014b (MathWorks). Standard preprocessing steps included bias correction, segmentation, and spatial normalization. To optimize inter-subject registration, we warped each image to a template derived from 300 confirmed neurologically healthy older adults (ages 44–86, M ± SD: 67.2 ± 7.3; 113 males, 186 females) scanned with one of three magnet strengths (1.5T, 3T, 4T), using affine and non-linear transformations with the help of the Diffeomorphic Anatomical Registration Through Exponentiated Lie Algebra (DARTEL) toolbox 23. Spatially normalized, segmented, and modulated gray matter images were smoothed using an 8-mm FWHM isotropic Gaussian kernel. Patient maps were compared to 534 confirmed neurologically healthy older adults from the UCSF MAC Hillblom Cohort (age range 44–99 years, M ± SD: 68.7 ± 9.1; 220 male/302 female), adjusted for age, sex, total intracranial volume, and magnet strength. W-scores are interpreted like z-scores, with *M* = 0/SD = 1. Negative w-scores represent below-average volume and scores <−1.50 fall below the 7th percentile and are thus considered clinically abnormal (La Joie et al., 2012; Ossenkoppele et al., 2015; Toller et al., 2021). To visualize variant-specific atrophy distribution, the average w-map for each variant was computed. The expected patterns of atrophy are shown in Figure 1A.

## Results

**FIGURE 2:**
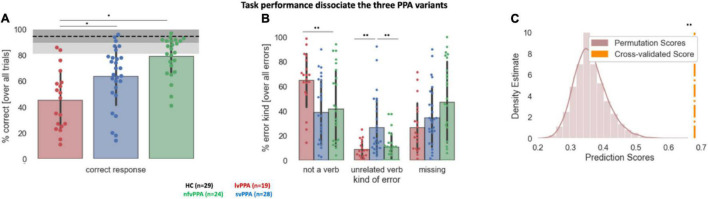
Behavioral performance during the auditory verb generation task. **(A)** Overall accuracy (% correct responses) across the three PPA variants. The dotted line denotes healthy controls (HC) average, the dark gray shaded area HC standard deviation, and the light gray area the full range of HC data. **(B)** Percentage of each error type across the three PPA variants. Error bars indicate standard deviation. Each dot represents a patient. Asterisk(s) denote(s) significant differences at **p < 0.01, *p < 0.05. **(C)** Results of the machine learning algorithm trained to classify the participants in the correct diagnostic group. The density distribution of the permutation scores is shown in light brown, the cross-validated score in orange. **Denotes the significant cross-validated score (*p* < 0.001).

Task performance in the auditory verb generation task is displayed in Figure 2. First, overall accuracy statistically differed across cohorts [*F*(3) = 34.06, *p* < 0.001], with *post hoc* analyses showing that all pairwise differences were statistically significant (all *ps* < 0.05 FWER, Figure 2A). *Post hoc* analyses indicated that accuracy was highest for nfvPPA and least accurate in lvPPA (Figure 2A). In contrast to accuracy, reaction times across the three variants were not significantly different [*F*(2) = 2.23, *p* = 0.115; all pairwise comparisons *p* > 0.09; lvPPA: mean = 2.20, STD = 0.47; svPPA: mean = 2.03, STD = 0.59; nfvPPA: mean = 1.86, STD = 0.40].

Second, the comparison of the three variants with respect to their error pattern revealed a main effect of group [*F*(2) = 13.80, *p* < 0.001], a main effect of error type [*F*(2) = 26.27, *p* < 0.001], and their interaction effect [*F*(4) = 10.50, *p* < 0.001]. While analyses of missing answers indicated no significant difference between cohorts, lvPPA produced statistically more not-a-verb responses compared to nfvPPA or svPPA (*p* = 0.001), and svPPA produced statistically more unrelated verb responses compared to nfvPPA or lvPPA (*p* = 0.001) (Figure 2B). All three PPA cohorts produced a similar amount of error consistency across each noun stimulus presentation: svPPA 34.06 ± 8.8; lvPPA 35.25 ± 8.15, nfvPPA 28.44 ± 11.74 (*p* > 0.05 with none of the group comparisons surviving multiple comparisons correction). Similarly, no difference emerged in terms of error type consistency: svPPA 68.00 ± 22.51; lvPPA 70.06 ± 17.09, nfvPPA 63.18 ± 31.48. Average accuracy for each PPA variant and their performance, stratified by error type, are presented in Table 3. Finally, based on error patterns a decision tree classifier algorithm was able to classify the participants in the correct diagnostic group well above chance level (cross-validated score = 0.65 ± 0.07, *p* < 0.001, theoretical chance is 33.33) (Figure 2C).

**TABLE 3:** Behavioral performance across error types and PPA variants.

*Dx*	*mean*	*sd*	*range (min-max)*	*error type*	*mean*	*sd*	*range (min-max)*
lvPPA	45.1	23.1	11–86	*Not_verb*	37.9	21.2	2–76
				*Unr_verb*	3.9	3.0	6–9
				*Missing*	13.1	12.0	17–51
svPPA	63.9	22.8	14–96	*Not_verb*	17.3	19.6	0–72
				*Unr_verb*	7.6	7.0	0–28
				*Missing*	11.3	9.9	0–37
nfvPPA	79.2	15.2	41–97	*Not_verb*	7.8	7.9	0–32
				*Unr_verb*	2.1	2.5	0–9
				*Missing*	11.0	12.4	0–43

*Overall accuracy in the auditory verb generation task as well as mean, standard deviation (STD), and range (min-max) for each error type are reported across the PPA variants. Dx, diagnosis; Not_verb, “not-a-verb” error type; Unr_verb, unrelated verb error type; Missing, missed trial.*

**FIGURE 3:**
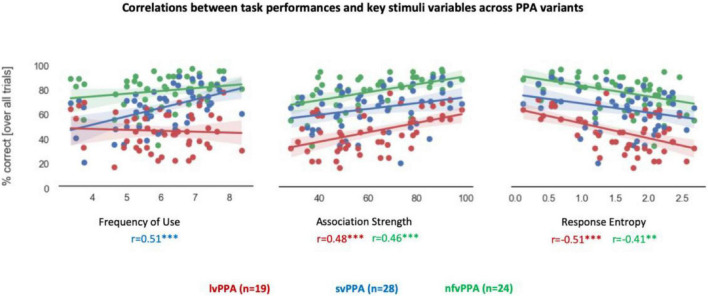
Correlations between performance and stimuli variables. Correlation coefficient values below each graph are color-coded by PPA variant. Asterisk(s) denote(s) significant correlation at ***p < 0.001, **p < 0.01. Correlations between percentage correct scores and lexical-semantics (Frequency of Use), retrieval demands (Association Strength), and overall agreement (Response entropy) are presented. Each dot represents a stimulus.

Associations between task performance and critical variables of the stimuli or neuropsychological measures are shown in Figure 3. There was a significant effect of word frequency (i.e., lexical aspect) in svPPA (*r* = 0.51, *p* < 0.001). No significant correlations were found with word length (i.e., word form aspect) in any variants, and svPPA was the only one showing a significant effect of semantic neighborhood density (*r* = 0.29, 0.0436), albeit not surviving multiple comparison corrections. In both lvPPA and nfvPPA, there was significant correlation between task performance and retrieval demands (i.e., ASI, lvPPA: *r* = 0.48, *p* < 0.001; nfvPPA: *r* = 0.46, *p* < 0.001). Similarly, the two variants also exhibited a significant effect of overall agreement (i.e., Response Entropy, lvPPA: *r* = −0.51, *p* < 0.001; nfvPPA: *r* = −0.41, *p* = 0.003). Competition demands (i.e., CSI) correlated with lvPPA performance but did not survive Bonferroni correction (*r* = 0.34, *p* = 0.015). Similarly, in svPPA, correlations with retrieval demands (*r* = 0.28, *p* = 0.045) and overall agreement (*r* = −0.3, *p* = 0.033) did not survive Bonferroni correction. No correlation survived correction when examining the three neuropsychological measures selected to assess word comprehension, lexical retrieval, and executive function. At the uncorrected level, a relationship was observed between task performance and executive functions (i.e., Modified Trails) in lvPPA (*r* = −0.53, *p* = 0.022) and nfvPPA (*r* = −0.45, *p* = 0.027).

**FIGURE 4:**
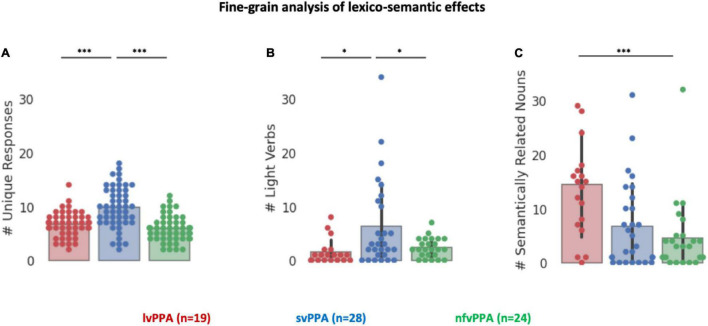
Fine-grained analysis of lexico-semantic effects. **(A)** Number of unique responses produced by each patient. **(B)** Number of light verbs produced by each patient. **(C)** Number of semantically related nouns produced by each patient. Asterisk denotes a significant correlation at ***p < 0.001, *p < 0.05.

Finally, the lexico-semantic analysis of the task responses highlighted three additional key differences between the three variants (Figure 4). First, svPPA produced more unique verbs (i.e., higher dispersion of the responses) than other clinical cohorts [*F*(2) = 25.40, *p* < 0.001], with *post hoc* tests indicating that svPPA patients are statistically different from both lvPPA and nfvPPA (adjusted-*ps* = 0.001), while these two cohorts did not differ from each other (adjusted *p* = 0.31). Second, the analysis of light verbs responses revealed a main effect of diagnosis [*F*(2) = 5.60, *p* = 0.005] and planned *post hoc* tests indicate that *svPPA* patients produced disproportionally *more light verbs* than the other two cohorts (8.69% against 1.86% in lvPPA and 2.31% in nfvPPA, adjusted-*ps* < 0.05), while these two cohorts did not differ from each other (adjusted *p* = 0.86). Third, the analysis of the number of semantically related nouns produced revealed a main effect of diagnosis [*F*(2) = 8.34, *p* < 0.001] and planned *post hoc* tests indicate that *lvPPA* patients produced disproportionally *more semantically related nouns* than both svPPA and nfvPPA (adjusted-*ps* < 0.005), while these two cohorts did not differ from each other (adjusted *p* = 0.60). No significant effect was found comparing the number of repetitions [*F*(2) = 1.62, *p* = 0.2].

## Discussion

In this study, we investigated whether performance on a single language task could dissociate PPA variants based on accuracy and the error patterns. To this end, we recruited a large sample of well-characterized PPA patients and asked them to perform an auditory verb generation task requiring manipulation of linguistic information at different processing stages: access to phonological word form (known to be affected in lvPPA), lexical-semantics (known to be affect in svPPA), and articulatory programming (known to be affected in nfvPPA). Although low performance on the task was observed across PPA variants, we observed variant-specific error patterns allowing syndromic classification, with task performance being associated with critical psycholinguistic features of the stimuli (e.g., word frequency in svPPA and noun-verb association demands in lvPPA and nfvPPA). These findings have important implications for clinical phenotyping and differential diagnosis of PPA variants, suggesting a breakdown at different stages of processing.

### Correct Responses Are All Alike: Every Error Is an Error in Its Own Way

Our investigation demonstrates that one single language task can help dissociate the three variants of PPA as long as attention is paid to the pattern of errors.

Patients with lvPPA had the lowest accuracy and produced the highest percentage of not-a-verb responses for a given noun (production of *baseball* instead of *throw* to a noun *ball*). The performance was affected by both retrieval demands (i.e., strength of the noun-verb association) and general noun-verb agreement (i.e., entropy of HC responses). Only a few studies have described and analyzed errors in lvPPA, and these studies mainly reported phonological errors (e.g., omissions, substitutions, or addition of sounds) across naming, fluency, repetition, and spontaneous speech tasks (Petroi et al., 2014, 2020; Henry et al., 2016; Dalton et al., 2018). Our study emphasizes category-specific error patterns such as the highest number of semantically related nouns (e.g., *baseball*) in lvPPA during a task that requires access to noun-verb combinations (e.g., ball-throw). This finding aligns well with previous studies implicating the IPL in event semantics and verb processing (e.g., Binder et al., 2009; Binder and Desai, 2011; Thompson and Meltzer-Asscher, 2014; see Lukic et al., 2021 for a comprehensive review across neurodegenerative disorders), a region commonly atrophied in lvPPA. Our study also showed that other factors may influence frequency of errors such as global cognition and stimuli demands, in line with others showing influence of overall aphasia severity and task complexity (Petroi et al., 2014).

Conversely, svPPA patients produce the highest percentage of unrelated-verb responses such as the highest number of light verbs (e.g., production of *do, take, get* in response to stimuli “laundry,” “ball,” and “cash,” respectively, instead of heavier verb responses such as *fold, throw, spend*). Unsurprisingly, their task performance correlated with lexical-semantic variables of the stimuli such as frequency of use. This is in line with previous studies showing that svPPA performance is influenced by familiarity and frequency during naming and that they have difficulty with retrieving semantically heavier (vs. lighter) verbs during a story completion task (e.g., Marcotte et al., 2014).

Finally, nfvPPA patient’s performance, while being the highest among the three variants, was still statistically different from that of HC. Like in lvPPA, task performance was affected by retrieval demands and general agreement. While not surviving corrections for multiple comparisons, correlations were also found between overall task performance and executive function. Executive deficits and increased rate of speech phonemic errors are often presented in nfvPPA and associated with frontal lobe integrity (e.g., Knibb et al., 2009). However, in the current study we accepted speech phonemic errors as correct responses since we were interested particularly in lexical-semantic knowledge impairments.

In summary, as previously suggested by Dalton et al. (2018) in the context of paraphasias, fine-grained qualitative analyses of the kind of error produced by PPA patients can improve phenotyping and diagnostic sensitivity. The auditory verb generation task chosen is fairly complex as it requires analyses of auditory input (target noun), and semantic processing, lexical retrieval, and finally selection and execution of speech. Nevertheless, only two of the 73 PPA patients recruited were unable to complete it (see outliers’ exclusion), and administration time required only 10 min. We thus believe that this task, provided careful selection of the stimuli and nuanced analysis of the response, would be helpful in deepening our understanding of speech-language errors and the neurocognitive characterization of PPA variants.

### A Window Into the Neurocognitive Basis of Language

Our findings from PPA suggest critical causal evidence of the role played by different left-hemisphere networks to language comprehension and production. In particular, relating specific error patterns to atrophy or hypometabolic patterns in relatively circumscribed brain areas, we corroborate the link between lexical processing and left superior and middle temporal gyri, and semantic processes and inferior and middle temporal gyri, and the ATL (see Catricalà et al., 2020).

In lvPPA patients, with left temporo-parietal cortical damage and anomia, heightened production of semantically related nouns suggests pronounced lexical-semantic deficits with an intact semantic system. This is in line with studies demonstrating that circumlocution errors are frequent in lvPPA (Budd et al., 2010; see Pressman and Gorno-Tempini, 2016) and post-stroke anomia (Halai et al., 2018). Although lvPPA and svPPA atrophy partially overlap in left middle and/or inferior temporal gyrus (Figure 1), the distinct patterns of errors suggest that lvPPA, unlike svPPA patients, fail to produce verbs due to temporo-parietal junction atrophy, corroborating evidence of a primary role of posterior perisylvian network in verb processing (Thompson et al., 2007, 2010; Lukic et al., 2021).

In svPPA patients, with anterior temporal lobe damage and semantic loss, heightened production of light verbs indicates preserved retrieval of semantically impoverished verbs. This finding corroborates models that emphasize the central role of semantic representations in heavy verbs and argues that light verbs are semantically less specific and may appear in various syntactic contexts (Gordon and Dell, 2003). The most consistent set of data that support this account comes from post-stroke aphasia (e.g., Breedin et al., 1998; Kim and Thompson, 2004; Barde et al., 2006). For instance, patients with post-stroke agrammatic aphasia showed greater impairment in production of semantically lighter verbs compared to heavier verbs during sentence completion and narrative task (Kim and Thompson, 2004; Barde et al., 2006). In contrast, previous studies showed that verb production is affected by semantic complexity in svPPA, but not in nfvPPA or lvPPA patients, in that svPPA showed greater impairment in production of semantically heavier verbs compared to lighter verbs on picture naming and verb story completion tasks (Méligne et al., 2011; Marcotte et al., 2014). These findings provide strong evidence for a heavy/light verb differential behavior if semantic and syntactic inputs are lesioned, respectively. Our findings in svPPA patients are in line with this prediction.

Finally, our findings on the noun-verb association variables corroborate growing evidence that association strength is a better predictor of performance than competition demands, when operationalized as agreement and ratio, respectively, but that neither measure is sufficient to fully explain behavioral results (e.g., see Snyder and Munakata, 2008).

### Limitations and Future Perspectives

One caveat of the current study is that we did not pre-select our stimuli to orthogonalize variables such as, for instance, selection vs. retrieval demands. Previous studies have debated on whether the behavioral effects observed during verb generation tasks are driven by competition demands (i.e., how difficult the selection of a particular verb is for a given noun, e.g., Thompson-Schill et al., 1997, 1998), or rather by retrieval demands (i.e., strength of the noun-verb association, e.g., Martin and Yan, 2006), ultimately suggesting that HC performance is affected by both variables simultaneously (Snyder and Munakata, 2008). Nevertheless, we attempted to disentangle these effects in our PPA variants as one could expect retrieval demands to be related with semantic performance and supported by temporal lobe structures (known to be affected by svPPA), while competition demands would be associated with executive functions and frontal lobe regions (known to be affected by nfvPPA). In our sample, neither competition demands, nor retrieval demands survived correction for multiple comparisons. Our choice of leveraging the variability offered by continuous measures (rather than dichotomous ones), prevents us from teasing apart specific contributions and limits our investigation of their underlying mechanisms.

Finally, our correlations between patients’ neuropsychological scores and task performance are limited by the fact that (1) not all crucial measures are available in all subjects, and (2) some neuropsychological tests are at ceiling (or floor) in patients. Similarly, the relatively low number of errors prevents us from running correlation analyses between psycholinguistic variables, neuropsychological performance and/or regional atrophy and specific kinds of errors. Future studies that utilize larger datasets and incorporate relevant stimuli to orthogonalize retrieval and selection demands will be better suited to explore the cognitive and neural bases of these error types.

## Conclusion

The findings of our experimental auditory verb generation task indicate that error patterns produced by patients with neurodegenerative disease are linked to the breakdown of different neurocognitive mechanisms involved in language processing, harbored in left hemisphere networks. Future neuropsychological and neuroimaging studies will be able to capitalize on this paradigm to further investigate these mechanisms and to elucidate the neural dynamics behind them.

## Data Availability Statement

The datasets presented in this article are not readily available because the sensitive nature of patients’ data and our current ethics protocol do not permit open data sharing. The clinical and neuroimaging data used in the current manuscript are available from SN, upon formal request indicating name and affiliation of the researcher as well as a brief description of the use that will be done of the data. All requests will undergo UCSF regulated procedure thus require submission of a Material Transfer Agreement (MTA) which can be found at https://icd.ucsf.edu/material-transfer-and-data-agreements. No commercial use would be approved. Requests to access the datasets should be directed to SN, srikantan.nagarajan@ucsf.edu.

## Ethics Statement

The studies involving human participants were reviewed and approved by the UCSF Committee for Human Research. The patients/participants provided their written informed consent to participate in this study.

## Author Contributions

EW, BR, RB, AW, and LH supported the data acquisition. ZM, AG, JH, SN, and MG-T provided analytical support and secured funding. SL, AL, and VB analyzed and interpreted the data and wrote the manuscript. All authors contributed to the article and approved the submitted version.

## Conflict of Interest

The authors declare that the research was conducted in the absence of any commercial or financial relationships that could be construed as a potential conflict of interest.

## Publisher’s Note

All claims expressed in this article are solely those of the authors and do not necessarily represent those of their affiliated organizations, or those of the publisher, the editors and the reviewers. Any product that may be evaluated in this article, or claim that may be made by its manufacturer, is not guaranteed or endorsed by the publisher.

## Footnotes

1
https://pstnet.com/products/e-prime/

2
https://scikit-learn.org

3
https://numpy.org

4
https://www.scipy.org

5
https://www.statsmodels.org
